# GradPose: a very fast and memory-efficient gradient descent-based tool for superimposing millions of protein structures from computational simulations

**DOI:** 10.1093/bioinformatics/btad444

**Published:** 2023-07-20

**Authors:** Daniel T Rademaker, Kevin J van Geemen, Li C Xue

**Affiliations:** Department of Medical BioSciences, Radboud University Medical Center, 6525 GA Nijmegen, The Netherlands; Department of Medical BioSciences, Radboud University Medical Center, 6525 GA Nijmegen, The Netherlands; Department of Medical BioSciences, Radboud University Medical Center, 6525 GA Nijmegen, The Netherlands

## Abstract

**Summary:**

Computational simulations like molecular dynamics and docking are providing crucial insights into the dynamics and interaction conformations of proteins, complementing experimental methods for determining protein structures. These methods often generate millions of protein conformations, necessitating highly efficient structure comparison and clustering methods to analyze the results. In this article, we introduce GradPose, a fast and memory-efficient structural superimposition tool for models generated by these large-scale simulations. GradPose uses gradient descent to optimally superimpose structures by optimizing rotation quaternions and can handle insertions and deletions compared to the reference structure. It is capable of superimposing thousands to millions of protein structures on standard hardware and utilizes multiple CPU cores and, if available, CUDA acceleration to further decrease superimposition time. Our results indicate that GradPose generally outperforms traditional methods, with a speed improvement of 2–65 times and memory requirement reduction of 1.7–48 times, with larger protein structures benefiting the most. We observed that traditional methods outperformed GradPose only with very small proteins consisting of ∼20 residues. The prerequisite of GradPose is that residue–residue correspondence is predetermined. With GradPose, we aim to provide a computationally efficient solution to the challenge of efficiently handling the demand for structural alignment in the computational simulation field.

**Availability and implementation:**

Source code is freely available at https://github.com/X-lab-3D/GradPose; doi:10.5281/zenodo.7671922.

## 1 Introduction

The field of structural bioinformatics heavily relies on protein structure comparisons. In the past decades, a variety of methods have been proposed for protein structure comparisons. They are designed mainly for two different scenarios: (i) where the equivalent positions of the structures to be compared are unknown, for example, experimental structures in the PDB databank and (ii) where the equivalent positions are already known, for example, models produced from molecular simulations. For clarity, we refer to the methods designed for the first scenario as “structural alignment algorithms” and those for the second scenario as “structural superimposition algorithms.”

Popular structural alignment algorithms include TM-align (Zhang and Skolnick 2005), which employs a dynamic programming approach to optimize the TM-score, a metric that reflects structural similarity; FATCAT (Ye and Godzik 2003), an approach that uses dynamic programming to find optimal chaining of aligned fragment pairs to allow flexible structure alignment; and CEalign (Shindyalov and Bourne 1998), a method based on the Combinatorial Extension algorithm that aligns protein structures by detecting common substructures. However, when equivalent positions are already known, as is often the case with structural models that generated by computational simulations such as molecular simulations, computational docking, or homology modeling, structural superimposition algorithms can be used instead. These algorithms have the potential to be faster and offer guarantees of finding optimal alignments, even when structures differ significantly, unlike the previously mentioned structural alignment methods, which are NP-hard (Ma and Wang 2014). Despite this advantage, computational simulations can still produce millions of structures, requiring fast and memory-efficient alignment methods to handle the vast amounts of data, especially when structures need to be aligned multiple times, for example, based on different regions of interest. There are several widely used software tools for performing structural superimposition, including ProFit (bioinf.org.uk—Prof. Andrew C.R. Martin’s group at UCL), ChimeraX (Goddard *et al.* 2018), SuperPose (Maiti *et al.* 2004), PyMOL, and PDB2SQL (Renaud and Geng 2020). These tools typically use a variation of one of two methods for generating the optimal rotation matrix to minimize the root mean squared deviation (RMSD) between protein structures: SVD (single value decomposition) of the covariance matrix approach (e.g. Kabsch 1976) or the quaternions method (e.g. Kearsley 1989). While both approaches can generate optimal superimpositions, they can be computationally intensive and require a significant amount of memory when processing large numbers of protein structures. Additionally, the presence of added or missing amino acids between structures can prevent these methods from fully leveraging efficient batch matrix operations for large datasets, leading to significantly longer alignment times.

We have developed GradPose, a new tool that tackles the challenges associated with structural superimposition. Designed for researchers who work with protein structures generated from computational simulations, this command-line tool uses gradient descent to optimize the rotation quaternions and superimpose multiple structures simultaneously. This new approach eliminates the need to calculate large covariance matrices, making it memory-efficient and scalable. By posing the problem as tensor operations, it can optimally utilize specialized libraries and hardware. Thanks to a masking procedure, GradPose can handle deletions compared to the reference with negligible added computation costs. Additionally, GradPose utilizes multiple CPU cores to optimize speed, and if a GPU is available, it can use CUDA acceleration to further improve performance. GradPose is user-friendly and provides a range of options. Overall, GradPose outperforms traditional methods in terms of speed, memory efficiency, and hardware options, while maintaining accuracy.

## 2 Methods

Preparing the structures: first, the GradPose algorithm extracts the 3D coordinates of each residue’s α-carbon from PDB files. It does this in batches of 50 000 (configurable) structures. After that, it generates a binary masking matrix to account for any deletions compared to the given reference structure. Then it standardizes the coordinates of the structures. Finally, it assigns a set of quaternions, randomly generated from a uniform distribution between 0 and 1, to each structure. The loading process is optimized by utilizing multiple CPU cores, with the I/O speed of the storage device being the primary limiting factor.

Performing structural superimposition: GradPose finds the optimal rotation quaternions by using standard gradient descent to minimize the RMSDs between the structures to be rotated and the reference structure. The optimization process consists of two phases: a rough and quick superimposition phase with a large step size, and a fine-tuning phase with a smaller step size to approach the optimal quaternions (see Supplementary material “Algorithms”). The use of a large initial step size in the first phase allows the algorithm to make rapid progress and explore a wide range of the rotation space, while decreasing the step size in the fine-tuning phase allows the algorithm to make finer adjustments and converge to the optimal solution. Empirically, we found that the length of the first phase depends on the number of residues selected, with a larger number of residues requiring fewer steps. The fine-tuning phase always has a fixed length to ensure complete convergence.

Applying the quaternions: once the optimal quaternions have been determined, the all-atom PDB files are read in and translated and rotated using the same center coordinate and quaternions from the preparation step. The resulting structures are saved to a given output folder. To accelerate the process of reading and writing to disk, the GradPose algorithm utilizes multiple CPU cores, as I/O to the storage device is the primary limiting factor.

## 3 Insights and benchmarks

Tools for structural superimposition include ProFit, ChimeraX, SuperPose, PyMOL, and PDB2SQL. PyMol and ChimeraX are mainly used for visualizing and analyzing proteins but are not optimized for fast and efficient superimposition. SuperPose is a robust online server tool with numerous options; nevertheless, the requirement to upload PDBs to the server limits its usability for big batch superimpositions. Additionally, it only returns the chains on which we align but no other chains. PDB2SQL is a tool that converts protein structure data from the PDB format into SQL commands for storing the data in a database. It is useful for researchers working with protein structure data and allows for easier access and manipulation of the data via SQL commands, but it does not contain the code to automatically do superimpositions of many structures. It is important to note that while these tools are capable of performing structural superimposition, they may not be optimized for it and may not be suitable for large batch analyses or high-throughput workflows. ProFit, however, was designed to perform large batch operations, which is why we choose it as the main comparison software for benchmarking.

We evaluated the performance of GradPose against ProFit (v3.3) using several simulated protein structure datasets, including structures generated by HADDOCK (Dominguez *et al.* 2003, van Zundert *et al.* 2016), a lagship docking software. For each tool and dataset, we recorded the maximum memory usage and the time taken from start to finish. Table 1 summarizes the results and shows that GradPose outperformed ProFit in terms of both memory usage and time, except for the 2jof_aug_1k dataset (Trp-cage miniprotein with 20 residues). This particular dataset was the only one we could find where ProFit was slightly faster due to the unusual small size of the protein. However, ProFit still required significantly more memory. In all other cases, GradPose was between 2 and 65 times faster and had 1.7 and 48 times lower memory usage compared to ProFit, with larger structures benefitting the most. Note that the memory usage of GradPose could be even further reduced with the “batch size” parameter. Detailed descriptions of the datasets and benchmarking procedures are found in Supplementary materials.

**Table 1.** Comparison of GradPose and ProFit runs on various simulated protein structure datasets, using 8 CPU cores.^a^

	GradPose			ProFit		
Dataset	Memory (GB)	Time (s)	Mean RMSD ± SD	Memory (GB)	Time (s)	Mean RMSD ± SD
*2jof_aug_1k*	0.18	10.6	1.13 ± 5.09 × 10^−1^	0.32	4.4	1.13 ± 5.10 × 10^−1^
*2jof_aug_10k*	0.23	33.1	1.15 ± 4.81 × 10^−1^	0.48	63.5	1.15 ± 4.81 × 10^−1^
*3b43_aug_1k*	0.18	13.9	1.39 ± 5.53 × 10^−1^	0.66	34.1	1.39 ± 5.53 × 10^−1^
*3b43_aug_10k*	0.57	84.5	1.41 ± 5.86 × 10^−1^	4.03	410.8	1.41 ± 5.86 × 10^−1^
*3vkg_aug_1k*	0.46	96.2	1.61 ± 6.67 × 10^−1^	4.17	4818.1	1.61 ± 6.67 × 10^−1^
*3vkg_aug_10k*	4.25	896.2	1.55 ± 6.36 × 10^−1^	41.76	58 694.2	1.55 ± 6.36 × 10^−1^
*1acb_docking_1k*	0.53	40.0	3.59 × 10^−1^ ± 1.60 × 10^−1^	2.12	166.5	3.59 × 10^−1^ ± 1.60 × 10^−1^
*1acb_docking_all*	0.99	396.8	7.15 × 10^−2^ ± 2.19 × 10^−1^	47.94	5294.9	7.15 × 10^−2^ ± 2.19 × 10^−1^
*1a1m_1k*	0.25	13.3	1.28 × 10^−4^ ± 3.34 × 10^−4^	2.35	219.0	1.26 × 10^−4^ ± 3.32 × 10^−4^
*1a1m_10k*	0.77	90.6	1.38 × 10^−4^ ± 3.45 × 10^−4^	21.23	705.0	1.44 × 10^−4^ ± 3.51 × 10^−4^
*1a1m_100k*	1.86	843.0	1.00 × 10^−3^ ± 4.47 × 10^−6^			
*1a1m_1mil*	2.77	14 088.9	1.00 × 10^−3^ ± 1.41 × 10^−6^			

a The table presents maximum memory usage, run time, and RMSDs after alignment. The RMSD values are included as a means of verifying the quality of the alignment, and any discrepancies between the algorithms would indicate errors in the alignment process. Some datasets in the table include synthetically generated homologs that are randomly rotated and translated before testing, indicated by the “_aug” suffix. PDB datasets have different origins (see “Datasets” in Supplementary material), and all RMSDs are calculated by a third-party algorithm, PDB2SQL, to avoid bias.

## 4 Conclusion and discussions

GradPose is a software tool that has the potential to advance the field of protein structure analysis and modeling. With the ability to superimpose millions of protein structures efficiently, GradPose allows researchers to perform large-scale structural comparisons and analyses that were previously computationally daunting. For example, superimposing millions of docking models to the target structure can now be done within minutes. GradPose assumes equivalent residues are numbered identically between structures. However, when working with structures that do not have correctly numbered residues, such as those generated by homology modeling, we recommend using multiple sequence alignments (Jia and Jernigan 2021, Rademaker *et al.* 2022) to establish equivalent positions between structures. Overall, GradPose has the potential to accelerate research in many areas of structural biology and contribute to the development of new therapies and drugs.

## Supplementary Material

btad444_Supplementary_Data
