# Supplementary material for: GradPose: a very fast and memory-efficient gradient descent-based tool for superimposing millions of protein structures from computational simulations
Source: Bioinformatics. 2023 Jul 20;39(8):btad444. doi: 10.1093/bioinformatics/btad444 (PMC10397417; doi:10.1093/bioinformatics/btad444)
Supplement: btad444_Supplementary_Data [file btad444_supplementary_data.pdf]

Supplementary document

GradPose: a very fast and memory-efficient gradient descent-based tool for superimposing millions of protein structures

Daniel T. Rademaker[1,†] , Kevin J. van Geemen[1,†] and Li C. Xue[1]

[1] Center for Molecular and Biomolecular Informatics, Radboud University Medical Center, Geert Grooteplein Zuid 26-28, 6525 GA Nijmegen, The Netherlands.

[†] Shared first authorship.

Table of Contents

1. Algorithms 3
   1.1 Standardizing Coordinates Algorithm 3
   1.2 Normalizing Quaternion Algorithm 3
   1.3 Converting Quaternions to Rotation Matrices Algorithm 4
   1.4 Structure Rotation Algorithm 5
   1.5 Quaternion Gradient Calculation for Structure Alignment Algorithm 5
   1.6 Main GradPose Algorithm 6
   1.7 PDB Conversion and Saving algorithm 7
2. GradPose Manual 8
   2.1 Dependencies 8
   2.2 Install 8
   2.3 Usage 8
   2.4 Example usages 8
3. Dataset information 10
   3.1 Dataset details 10
   3.2 Choice of PDBs for benchmarking 10
      2jof 10
      3b43 11
      3vkg 11
      1acb 11
      1a1m 11
   3.3 Augmentation of PDBs 11
   3.4 Benchmarking 11
   3.5 GPU benchmarking 12
4. GradPose Usage 13
   4.1 Materials 13
   4.2 Command-line usage 13
   4.3 Python usage 13
5. Supplementary References 15

1. Algorithms

1.1 Standardizing Coordinates Algorithm

The pseudocode in Algorithm 1 describes how to standardize the coordinates of structures after retrieving them from the PDB files. The algorithm takes in a set of coordinates (xyz) and performs the following steps:

1. Subtract the mean of the coordinates from each coordinate, effectively centering the coordinates around the origin.
2. Calculate the scale factor. This by taking a structures' coordinates, calculate the norm values of the coordinates, and then calculate the standard deviation of those norm values multiplied by 100.
3. Divide each coordinate by the scale factor, resulting in a set of standardized coordinates.
4. Return the standardized coordinates.

Standardizing coordinates will make it that all types of proteins, large or small, will have roughly the same coordinates variance which has a stabilizing effect on the optimization process. Note that the normalized sizes of the structures do not matter as the optimization process is only concerned with finding the correct rotations which are independent of actual structure sizes.

Algorithm 1 Standardizing Coordinates Algorithm

```
def standardize_coordinates(xyz):
1: xyz = xyz – mean(xyz)
2: scale_factor = standard_deviation(xyz) x 100
3: xyz = xyz / scale_factor
4: return xyz
```

1.2 Normalizing Quaternion Algorithm

The pseudocode in Algorithm 2 describes the process of normalizing quaternion (q) values. The algorithm takes in a set of quaternions and performs the following steps:

1. Divide each quaternion by its vector norm, which is defined as the square root of the sum of the quaternion parts squared. This step ensures that the quaternion is a unit quaternion.
2. Return the normalized quaternions.

It's important to note that the quaternions are firstly initialized randomly from a uniform distribution between 0 and 1. And any update to the quaternions must be followed by this algorithm or otherwise it will not be a true unit quaternion.

Algorithm 2 Normalizing Quaternion Algorithm

```
def normalize_q(q):
1: q  = q  / vector_norm(q)
2: return q
```

1.3 Converting Quaternions to Rotation Matrices Algorithm

The pseudocode in Algorithm 3 describes the process of converting quaternions into rotation matrices. This is necessary because rotation matrices are required to rotate structures and align them with a reference structure. The algorithm takes in a set of quaternions (q) and performs the following steps:

1. All quaternion vectors are normalized to make sure they are true unit vectors.
2. For each quaternion vector in the input set (q), it creates a matrix (m)
3. through 3:12 it assigns values to each element of the matrix m following the mathematical expressions mentioned in the pseudocode.
4. It collects all created matrices in a tensor called matrices in the pseudocode.
5. It returns the tensor of matrices.

Note that in the real python code this is not done with a for loop, but more efficiently as tensor operations.

Algorithm 3 Converting Quaternions to Rotation Matrices Algorithm

```
def calc_rotation_matrices_from_quaternions(q)
1: q = normalize_q(q)
2: for all q_vectors in q, make matrix m:
3:    m[0,0] = (2  x  (q[0]^2 + q[1]^2)) - 1
4:    m[0,1] =  2  x  (q[1]  x q[2] - q[0] x q[3])
5:    m[0,2] =  2  x  (q[1]  x q[3] + q[0] x q[2])
6:    m[1,0] =  2  x  (q[1]  x q[2] + q[0] x q[3])
7:    m[1,1] = (2  x  (q[0]^2 + q[2]^2)) - 1
8:    m[1,2] =  2  x  (q[2]  x q[3] - q[0] x q[1])
9:    m[2,0] =  2  x  (q[1]  x q[3] - q[0] x q[2])
10:   m[2,1] =  2  x  (q[2]  x q[3] + q[0] x q[1])
11:   m[2,2] = (2  x  (q[0]^2 + q[3]^2)) - 1
12: end for
```

```
matrices = [m_1, m_2, … m_n]
return matrices
```

1.4 Structure Rotation Algorithm

The pseudocode in algorithm 4 describes the process of applying rotations to the coordinates of the structures. The algorithm takes in the coordinates of the structures (xyz) and a set of quaternions (q) as input. It performs the following steps:

1. It first calls the algorithm "calc_rotation_matrices_from_quaternions" passing the quaternions as input, this algorithm will return a tensor of rotation matrices.
2. It then calculates the dot product between the coordinates (xyz) and the rotation matrices, resulting in the rotated coordinates of the structures.
3. Finally, it returns the rotated coordinates of the structures.

This algorithm is used in the process of aligning structures to a reference structure by applying the rotations calculated in the previous steps to the structure. It is important to note that the dot product is used here as it is a standard method for applying rotations represented by matrices to coordinates of structures.

Algorithm 4 Structure Rotation Algorithm

```
def rotate_coordinates(xyz, q):
rotation_matrices = calc_rotation_matrices_from_quaternions(q)
rotated_xyz = dot_product(xyz, rotation_matrices)
return rotated_xyz
```

1.5 Quaternion Gradient Calculation for Structure Alignment Algorithm

The pseudocode in algorithm 5 describes an algorithm that compares the coordinates of a set of structures to a reference structure, calculates gradients for the quaternion values, and applies those gradients. The algorithm takes in the coordinates of all structures (xyz) and the reference structure coordinates (ref_xyz) as input. It performs the following steps:

1. It first calculates the mean squared error (MSE) between the coordinates of each structure and the reference structure coordinates.
2. It removed the MSE values between equivalent positions where the structures have deletions compared to the reference. It does this by multiplying the MSE values matrix with a binary matrix where deletions are denoted with a ‘False’ value.
3. It then calculates the gradients for the quaternion values using the sum of calculated MSE.
4. Finally, it applies the gradients to the quaternion values, updating them for the next iteration of the alignment process.

Algorithm 5 Quaternion Gradient Calculation for Structure Alignment

```
def take_update_step(q, learning_rate, xyz, reference_xyz):
rotated_xyx = rotate_coordinates(xyz, q)
loss_error_matrix = (reference_xyz - rotated_xyx)^2
loss_error = sum(loss_error_matrix x binary_matrix_with_deletions)
gradients_q = calculate_gradients(error)
q = q – learning_rate x gradients_q
return q
```

1.6 Main GradPose Algorithm

The pseudocode in algorithm 6 describes the optimization process for determining the quaternion values that align the structures to the reference structure. The algorithm uses different learning rates for its two phases of alignment. The first phase, which uses a learning rate of 100 and a number of steps determined by the formula max(75, -4*nmb_residues + 400), is intended to quickly bring the quaternions close to an optimal solution. This is done by taking large steps towards the optimal solution, which allows the algorithm to converge faster. The second phase, which uses a learning rate of 0.00001 and 25 steps, is intended to fine-tune the quaternions and reach the optimal solution. To prevent the unnormalized quaternion values from becoming excessively large, normalization is applied after each update step during the first phase of optimization.

Algorithm 6 Main GradPose Algorithm

```
def optimize_q(q, xyz, nmb_positions, xyz, reference_xyz):
big_update_steps = max(75, -4 x nmb_positions + 400)
big_learning_rate = 10e2
small_update_steps = 25
small_learning_rate = 10e-4
for step in number of big_update_steps:
   take_update_step(q, big_learning_rate, xyz, reference_xyz)
   q = normalize(q)
end for
for step in number of small_update_steps:
   take_update_step(q, small_learning_rate, xyz, reference_xyz)
end for
return q
```

1.7 PDB Conversion and Saving algorithm

The pseudocode in algorithm 7 describes the final step of the GradPose algorithm, where the full PDB files are read in, translated and rotated using the same centre coordinates and optimized quaternions from the preparation steps. The resulting structures are then saved to a specified output folder.

Algorithm 7 PDB Conversion and Saving

```
def convert_pdbs(pdb_files, q, output_folder):
for all pdb_files:
    xyz = read_coordinates(pdb)
    rotated_xyz = rotate_coordinates(xyz, q)
    save_new_pdb(pdb, output_folder, rotated_xyz)
end for
```

2. GradPose Manual

2.1 Dependencies

GradPose requires Python 3 to be installed on your system.

2.2 Install

Install GradPose using Python's package installer pip:

```
pip install gradpose
```

2.3 Usage

```
gradpose [-h] [-i INPUT] [-s] [-t REFERENCE] [-o OUTPUT] [-c CHAIN] [-r RE
SIDUES [RESIDUES ...]] [-n N_CORES] [-b BATCH_SIZE] [--gpu] [--silent] [--
verbose] [--rmsd]
```

Help and defaults for each argument can be viewed by executing GradPose with the help argument: `gradpose -h`. Alternatively, you can examine the example usages listed below.

2.4 Example usages

2.4.1 Input

To use GradPose, specify a folder folder containing any amount of PDBs to be used for alignment using the `-i` argument. For example, let's use a folder named 'example_folder'.

```
gradpose -i example_folder
```

or

```
gradpose example_folder
```

Note: Omitting `-i` is only possible if no other arguments are used. Note: The aligned proteins are automatically stored in the folder 'output'.

2.4.2 Output

Using another folder name, or overwriting the current folder without creating a second is possible using the `-o` argument.

```
gradpose -i example_folder -o example_output_folder
```

Note: To overwrite the current files, provide the same input and output folder.

2.4.3 REFERENCE

If the PDBs in folder 'example_folder' need to be superimposed to a specific reference structure, use the `-t` argument:

```
gradpose -i example_folder -t example_folder/reference_example.pdb
```

Note: The reference structure **does not** need to be in the same folder as the PDBs used for alignment.

2.4.4 Chain

By default, GradPose aligns to the longest chain in the reference PDB. You can choose a chain ID with the `-c` argument. In this example, the alignment is done on all residues of chain B:

```
gradpose -i example_folder -c B
```

2.4.5 Residues

By default, GradPose aligns to all residues of the selected chain. If a finer selection can be made with the `-r` argument. For example, to align on the first 10 residues, residues 12 and 14, and the residues ranging between (and including) 20 and 30 of chain B:

```
gradpose -i example_folder -c B -r 1:10 12 14 20:30
```

2.4.6 CPU Cores

By default, GradPose utilizes all CPU cores on the system. To manually specify the amount of cores, use `-n`.

```
gradpose -i example_folder -n 4
```

2.4.7 Batch Size

By default, the batch size is set to 50,000 to limit memory usage. To set a lower batch size, use the -b argument.

```
gradpose -i example_folder -b 1000
```

2.4.8 CUDA Acceleration

GPU acceleration is disabled by default. If you have a PyTorch installation with CUDA enabled, simply add the `--gpu` flag to the command to enable GPU acceleration.

```
gradpose -i example_folder --gpu
```

2.4.9 RMSD Calculation

GradPose can automatically calculate the RMSD of the residues on which it aligns compared to the reference structure for every PDB. This feature can be enabled using the `--rmsd` flag. The results will be saved as 'rmsd.tsv' in the output folder.

```
gradpose -i example_folder --rmsd
```

2.4.10 Verbosity Levels

GradPose allows the user to choose to run the tool silently, without generating any output in the console, with the `--silent` flag.

```
gradpose -i example_folder --silent
```

Alternatively, more verbose console output may be enabled with `--verbose`.

```
gradpose -i example_folder --verbose
```

3. Dataset information

3.1 Dataset details

Table 1. Dataset details.

NAME	PDB	AUGMENTED	NO. MODELS	NO. RESIDUES	NOTE
**2JOF_AUG_1K**	2jof	Yes	1000	20	Small protein
**2JOF_AUG_10K**	2jof	Yes	10,000	20	Small protein
**3B43_TITIN_AUG_1K**	3b43	Yes	1000	569	Long protein
**3B43_TITIN_AUG_10K**	3b43	Yes	10,000	569	Long protein
**3VKG_AUG_1K**	3vkg	Yes	1000	5807	Huge protein
**3VKG_AUG_10K**	3vkg	Yes	10,000	5807	Huge protein
**1ACB_DOCKING_1K**	1acb	No	1000	304	Docking models (randomly selected from _all)
**1ACB_DOCKING_ALL**	1acb	No	25300	304	Docking models
**1A1M_1K**	1a1m	Partially	1000	386	Only rotated and translated
**1A1M_10K**	1a1m	Partially	10,000	386	Only rotated and translated
**1A1M_50K**	1a1m	Partially	10,000	386	Only rotated and translated
**1A1M_100K**	1a1m	Partially	100,000	386	Only rotated and translated. Only for GradPose.
**1A1M_500K**	1a1m	Partially	10,000	386	Only rotated and translated
**1A1M_1MIL**	1a1m	Partially	1,000,000	386	Only rotated and translated. Only for Gradpose.

3.2 Choice of PDBs for benchmarking

Specific protein PDBs were chosen for benchmarking based on specific characteristics to compare normal and extreme cases for protein superimposition.

2jof

The Trp-cage: Optimizing the Stability of a Globular Miniprotein
10.2210/pdb2JOF/pdb
Residue count: 20
This protein was chosen for its small size. It is unlikely that researchers would need to align on a protein smaller than this, so it was selected to benchmark the lower limit of superimposition tools.

3b43
I-band fragment I65-I70 from titin
10.2210/pdb3B43/pdb
Residue count: 569
This protein was chosen for being very long in 3D space, not in the number of residues.

3vkg
X-ray structure of an MTBD truncation mutant of dynein motor domain
10.2210/pdb3VKG/pdb
Residue count: 5807
This protein was chosen for its large number of residues, to test the upper limit of superimposition tools.

1acb
CRYSTAL AND MOLECULAR STRUCTURE OF THE BOVINE ALPHA-CHYMOTRYPSIN-EGLIN C COMPLEX AT 2.0 ANGSTROMS RESOLUTION
10.2210/pdb1ACB/pdb
Residue count: 304
This protein was chosen to include a dataset from an existing docking models dataset. The docking was performed using HADDOCK (Dominguez et al., 2003) for the DeepRank paper (Renaud et al., 2021). This PDB was chosen because the docking dataset is an example of the kind of models that superimposition would be used for.
Dataset DOI: 10.15785/SBGRID/843

1a1m
MHC CLASS I MOLECULE B*5301 COMPLEXED WITH PEPTIDE TPYDINQML FROM GAG PROTEIN OF HIV2
10.2210/pdb1A1M/pdb
Residue count: 386
This protein was chosen as a good general example for use with superimposition.

3.3 Augmentation of PDBs
In addition to the docking models, we created datasets of artificial homologs. This was done in two steps. First, secondary structures were detected by the tool DSSP (Touw et al., 2014) and each secondary structure was slightly rotated by a random small 3D-rotation. Then, each PDB was given a random 3D-rotation and a small translational offset. We omitted step one for 1a1m as our primary goal for this protein was to evaluate the superimposition speed for huge batches.

3.4 Benchmarking
The benchmark cluster system specifications of the paper: Dell R810, 4x8 cores, 336GB RAM. Each benchmark was assigned 8 cores of the CPU.

ProFit v3.3 (Martin, 2020) was rebuilt from source for Linux to set the MAXSTRUC value to 1000000.

GradPose and ProFit were instructed to superimpose the chain A in each dataset. The reference structure was randomly selected but the same for both programs. Time was measured from the moment the process was launched and ended when it closed. Memory was constantly measured while the program ran, with the highest amount of memory saved. RMSDs were calculated after superimposition by PDB2SQL (Renaud & Geng, 2020). This eliminated any bias that either tool might have had when calculating the RMSDs themselves.

3.5 GPU benchmarking

For the GPU benchmarking, only presented here in the supplementary document, we used a standard personal computer system with specifications: Intel® Core™ i9-10850K CPU @ 3.60GHz × 20, 62.7 GiB RAM, and a NVIDIA GEFORCE RTX 3090 GPU. Each benchmark was assigned 20 cores of the CPU.

Table 2 summarizes the benchmarking results. All residues were included in the analysis. It's important to note that the times reported here only pertain to the alignment process (not including reading/writing of PDB files). An example with the complete timing information, including reading and writing of PDB files, can be found in Section 4 (GradPose Usage).

Table 2. Benchmarking of CPU vs GPU. Time is given in rounded seconds.

Dataset	CPU (s)	GPU (s)	CPU/GPU ratio
1a1m_10K	3	1	3.0
1a1m_50K	20	3	6.7
1a1m_100K	36	6	6.0
1a1m_500K	178	32	5.6
1a1m_1MIL	366	60	6.1

Note that the GPU is consistently roughly 6 times faster than the CPU, except on the smallest dataset. We assume this has to do with a fixed time it takes loading the data from the RAM into the GPU memory, which only dominates at small sets.

4. GradPose Usage

4.1 Materials

The tool is implemented entirely in Python and utilizes the NumPy (1.19.5), PyTorch (1.13.0), and tqdm (4.64.0) libraries. The use of the torch library allows for optional CUDA acceleration if a device with CUDA support is available.

4.2 Command-line usage

An example of command line usage:

```
$ gradpose -i PDBs -t PDBs/1AGD.pdb -o aligned_PDBs -c M -r 1:180
```

This command line will produce the following example output:

```
=== Superimpose ===

Processing batch 1/1

No chain selected, using longest chain: M

Extracting backbones: 100%|██████████| 50001/50001 [00:04<00:00, 10393.77it/s]

Aligning backbones: 100%|██████████| 100/100 [00:15<00:00, 6.36it/s]

Rotating PDBs: 100%|██████████| 50000/50000 [00:40<00:00, 1249.33it/s]

=== Superimposing completed in 61.7s ===
```

Note that the 50,000 files required 4 seconds on a personal computer to load, 15 seconds to find the optimal rotations, and 40 seconds to process all PDB files, which equates to roughly 806 structures per second.

4.3 Python usage

```
import glob

import gradpose


def main():

    # Make a list of all PDB files to align.

    pdb_files = glob.glob("PDBs/*.pdb")


    # Superimposition with GradPose.
```

```
    gradpose.superpose(
        pdbs_list=pdb_files,
        reference="PDBs/1AGD.pdb",
        output="superimposed_PDBs",
        chain="M",
        residues=range(1, 181)  # 1 through 180
    )

if __name__ == "__main__":
    main()
```

5. Supplementary References

Dominguez, C., Boelens, R., & Bonvin, A. M. J. J. (2003). HADDOCK: A Protein−Protein Docking Approach Based on Biochemical or Biophysical Information. *Journal of the American Chemical Society*, 125(7), 1731–1737. https://doi.org/10.1021/ja026939x

Martin, A. C. R. (2020, April 27). *bioinf.org.uk - Prof. Andrew C.R. Martin's group at UCL*. http://www.bioinf.org.uk/software/profit/

Renaud, N., & Geng, C. (2020). The pdb2sql Python Package: Parsing, Manipulation and Analysis of PDB Files Using SQL Queries. *Journal of Open Source Software*, *5*(49), 2077. https://doi.org/10.21105/joss.02077

Renaud, N., Geng, C., Georgievska, S., Ambrosetti, F., Ridder, L., Marzella, D. F., Réau, M. F., Bonvin, A. M. J. J., & Xue, L. C. (2021). DeepRank: a deep learning framework for data mining 3D protein-protein interfaces. Nature Communications, 12(1). https://doi.org/10.1038/s41467-021-27396-0

Touw, W. G., Baakman, C., Black, J., te Beek, T. A., Krieger, E., Joosten, R. P., & Vriend, G. (2014). A series of PDB-related databanks for everyday needs. *Nucleic Acids Research*, *43*(D1), D364–D368. https://doi.org/10.1093/nar/gku1028
